# 

**Published:** 1914-09-12

**Authors:** 


### Medical Appointments.

Mr. J. Campbell Neil, M.B., Ch.B., Edin., has been appointed junior house surgeon to the Blackburn and East Lancashire Infirmary.

Dr. Holroyd, assistant county medical officer of health to Hereford, has been appointed assistant medical officer and school medical officer to the city of Norwich.

Mr. W. L. Holyoak, M.D., B.S., late clinical assistant Royal Westminster Ophthalmic Hospital, has been appointed ophthalmic house surgeon and resident officer to medical out-patients at the Leicester Royal Infirmary.

Dr. Charles P. M. Joubert has been appointed house surgeon to the Northern Infirmary, Inverness. A South African by birth, he graduated at Edinburgh University. He was in Berlin when the war broke out, and had an arduous journey home.

Dr. Montagu Travers Morgan, M.B., Ch.B., D.P.H., has been appointed assistant county medical officer of health to Hereford. He has held several resident appointments in Liverpool institutions, and graduated at Liverpool University.

The following appointments have recently been made at Ancoats Hospital, Manchester :—House surgeon : F. B. Smith, M.A., B.C. (Cantab.), M.R.C.S., L.R.C.P. House physician : F. S. Charnock, M.B., Ch.B., M.R.C.S., L.R.C.P. Assistant house surgeon : E. H. Walker, M.B., Ch.B.

Dr. Gray, assistant medical officer at the Bradford Municipal Tuberculosis Dispensary, having been called up for service on the medical staff of the War Office, Dr. Bryne, who had resigned the appointment, has complied with the Corporation's request to continue to perform the duties until the end of the war, his salary having been increased to £350 per annum.

### The Chair of Physiology at Sheffield.

The Council of the University of Sheffield has decided to invite Dr. J. B. Leathes, F.R.S., at present Professor of Pathological Chemistry in the University of Toronto, to accept the Chair of Physiology rendered vacant by the acceptance by Professor J. S. Macdonald of the Chair of Physiology in the University of Liverpool. Mr. Leathes graduated M.B., B.Ch. at Oxford University in 1893.

### A Founder of the Pharmacists' Association Retires.

Mr. W. E. Miller, M.P.S., pharmacist at the South Dispensary, Somers Town, N.W., has tendered his resignation to the St. Pancras Guardians, in whose service he has been for thirty-seven and a half years. In applying to have a sufficient time added to this period to make the maximum of forty years—*i.e.*, a retiring allowance of two-thirds of the average yearly salary for the last five years —Mr. Miller points out that by manufacturing most of the preparations required he has saved the Guardians a good deal of expenditure. The matter has been referred to the Finance Committee, who will, it is hoped, grant Mr. Miller's request.

Mr. Miller was one of the original founders of the Public Pharmacists' Association, having been on the council of that body for many years. The committee meetings have also been held at the South Dispensary, without interruption, ever since the formation of this body representing institutional pharmacists.

**RATES OF SUBSCRIPTION (Payable in advance).**

| *United Kingdom.* | | *Foreign and Colonial.* | |
|---|---|---|---|
| Three Months ... ... | 2s. 0d | Three Months ... ... | 2s. 6d. |
| Six Months ... ... | 4s. 0d | Six Months ... ... | 5s. 0d. |
| Twelve Months ... ... | 6s. 6d | Twelve Months ... ... | 9s. 6d. |

RATES OF SUBSCRIPTION: Twelve months, 6/6; Six months, 4/-; Three months, 2/-, post free to any address in the United Kingdom. Abroad, Twelve months, 8/8; Six months, 5/-; Three months, 2/6, post free.—Address The Subscription Dept., "THE HOSPITAL," The Hospital Building, 28/29 Southampton Street, Strand, London W.C.

